# Seropositivity among Korean Young Adults Approximately 2 Years after a Single-Dose Vaccination against Hepatitis A Virus

**DOI:** 10.1371/journal.pone.0142297

**Published:** 2015-11-05

**Authors:** Yeong-Jun Song, Jiseun Lim, Woong-Sub Park, Haesook Sohn, Moo-Sik Lee, Dong-Hoon Shin, Chun-Bae Kim, Hwasung Kim, Gyung-Jae Oh, Moran Ki

**Affiliations:** 1 Department of Preventive Medicine, Eulji University School of Medicine, Daejeon, Korea; 2 Department of Preventive Medicine & Public Health, College of Medicine, Kwandong University, Gangneung, Korea; 3 Department of Preventive Medicine, School of Medicine, Inje University, Busan, Korea; 4 Department of Preventive Medicine, College of Medicine, Konyang University, Daejeon, Korea; 5 Department of Preventive Medicine, School of Medicine, Keimyung University, Daegu, Korea; 6 Department of Preventive Medicine, Institute for Poverty Alleviation & International Development, Yonsei University Wonju College of Medicine, Wonju, Korea; 7 Department of Preventive Medicine, Medical College Soonchunhyang University, Cheonan, Korea; 8 Department of Preventive Medicine, Wonkwang University Medical School, Iksan, Korea; 9 Department of Cancer Control and Policy, Graduate School of Cancer Science and Policy, National Cancer Center, Goyang, Korea; Yonsei University College of Medicine, REPUBLIC OF KOREA

## Abstract

We previously observed 80.7% seropositivity and a significant interaction between gender and hepatitis A virus (HAV) vaccine type (Havrix vs. Epaxal) on the seropositivity approximately 11 months after single-dose HAV vaccinations in Korean young adults. Our objective was to evaluate seropositivity approximately 2 years after a single-dose HAV vaccination and the influence of demographic characteristics on seropositivity, including the interaction between gender and vaccine type. Seronegative medical school students were randomly vaccinated with Havrix or Epaxal. Based on a total serum anti-HAV antibody titer cutoff of 20 IU/mL, 338 participants (76.0%) of the 445 vaccinees were seropositive 20–25 months after a single-dose HAV vaccination. The seropositive rates were similar after vaccination with Havrix (77.0%) and Epaxal (74.9%). Univariate analysis indicated that female (*p* = 0.052) and less obese (*p* < 0.001) participants had a higher seropositive rate, whereas other characteristics such as age, alcohol use, smoking history, vaccine type, and follow-up duration were not associated with seropositivity. Multivariate analysis indicated that women (*p* = 0.026) and participants with moderate alcohol use (*p* < 0.001) showed significantly higher seropositive rates than men and participants with no or low alcohol use, respectively. The seropositive rates after vaccination with Havrix and Epaxal were 70.9% and 67.5% in men and 87.7% and 91.3% in women, respectively (*p* for interaction = 0.304). Compared with the seropositive rate approximately 11 months after vaccination, the seropositive rate decreased substantially only in men in the Havrix group (11.0% points), and consequently, the interaction between gender and vaccine type disappeared while seropositivity remained high (87.7% and 91.3% in Havrix and Epaxal groups, respectively) among women approximately 2 years after vaccination. Further studies are needed to assess whether the seropositive rate would be maintained in all groups more than 2 years after a single-dose HAV vaccination.

## Introduction

Although hepatitis A virus (HAV) infection is recognized as a vaccine-preventable disease, the introduction of a national hepatitis A immunization program faces several barriers including high vaccine prices and complex vaccination schedules [1]. Therefore, evidence of long-term immunity after a single-dose vaccination against HAV is necessary. However, previous studies have reported various results, ranging from 44.4% to 97.8% approximately 2 years after a single-dose HAV vaccination in adults. Furthermore, the ranges of follow-up duration after the vaccination of each study were so wide that there is a lack of information about the seropositivity at the time point of 2 years after the vaccination, and the effects of demographic characteristics other than gender have not been evaluated in most previous studies.

In our previous study, we found that immunogenicity differed according to the vaccine type only among men (81.9% vs. 69.2% in the Havrix and Epaxal groups, respectively), whereas the immunogenicity of both vaccines was similarly high among women (90.1% vs. 92.9% in the Havrix and Epaxal groups, respectively) [2]. This gender-vaccine type interaction has a clinical significance because if Epaxal consistently shows lower immunogenicity particularly in men, Havrix rather than Epaxal should be administered to men. It is also important to assess whether the high level of seropositivity will be maintained in women beyond 2 years, because if so, a single-dose strategy can be considered for a national HAV vaccination program in women.

We performed this study to evaluate seropositivity approximately 2 years after a single-dose HAV vaccination and the influence of demographic characteristics on seropositivity, including the interaction between gender and vaccine type observed in our previous study.

## Materials and Methods

Similar to our previous study [2], the current study was executed in the following eight medical schools located throughout the country: Eulji University, Kwandong University, Inje University, Konyang University, Keimyung University, Yonsei Wonju University, Soonchunhyang University, and Wonkwang University. Participants were enrolled and vaccinated from November 4, 2010 to April 7, 2011, and they were followed up from November 16, 2012 to December 14, 2012. The protocols of the study, including questionnaires were approved by the Institutional Review Board (IRB) of Eulji University (approval number: 10–69). Additionally, the IRBs of three medical schools reviewed and approved the study protocols (the approval numbers for Inje University, Keimyung University, and Yonsei Wonju University were 10–178, 10–155, and 2010–49, respectively). All IRBs had approved the study protocols before enrollment of participants began, and written informed consent was obtained from adult participants (n = 454) and the parents of participants under age 19 (n = 128).

### Study Group

Among the target population of 726 freshman or sophomore students from eight medical schools, 582 (80.2%) agreed to participate in this study. Their HAV seropositivity was tested using immunoassays, and they were asked to complete a questionnaire about demographic characteristics, weight and height, and previous vaccination history.

After excluding participants with previous HAV vaccination history or with HAV seropositivity, 483 participants were randomly allocated to be vaccinated with a single dose of either Havrix or Epaxal.

Among the 483 vaccinees, 450 participants (93.2%) could be monitored for approximately 2 years after vaccination. After excluding five participants who were individually vaccinated with a second dose, 445 participants (92.1%) were finally included in the data analysis. The 38 participants (7.9%) who dropped out or were excluded from the final analysis included 17 and 21 students vaccinated with Havrix and Epaxal, respectively. Their mean age was 19.6 (range 18–23) years. In addition, the female to male ratio (0.14) in this group was lower than that in the 445 participants included in the data analysis (0.51).

### Measuring Data

Serum anti-HAV antibody titers were measured by chemiluminescence using an immunoassay instrument (ADVIA Centaur; Siemens, Munich, Germany) [2]. Because the immunoassay equipment was set with 20 IU/mL as a cutoff value, we applied a cutoff of 20 IU/mL for total serum anti-HAV antibody titers for the assessment of HAV seropositivity. We considered an anti-HAV antibody titer of ≥ IU/mL as seropositive.

Obesity status was defined using body mass index (BMI) according to the guidelines of the International Obesity Task Force for Asian adults, as follows (in kg/m^2^): underweight, BMI <18.5; normal weight, BMI ≥18.5 and <23; overweight, BMI ≥23 and <25; and obese, BMI ≥25 [3]. Alcohol use was defined using the Korean version of the Alcohol Use Disorders Identification Test score (AUDIT-K) according to the guidelines of the World Health Organization, as follows: low, score of 0–7; medium, score of 8–15; high, score of 16–19; and very high, score of 20–40 [4, 5].

### Statistical Analysis

Seropositive rates across the subgroups were compared using the chi-square test, and trends across subgroups were analyzed using the linear by linear test. Logistic regression was used for multivariable analysis. All statistical analyses were performed using SPSS version 21 (IBM, Chicago, IL, USA).

## Results

Of the 445 vaccinees, 338 participants (76.0%) were seropositive 20–25 months after a single-dose vaccination against HAV. Similar seropositive rates were observed after vaccination with Havrix (77.0%) and Epaxal (74.9%). In univariate analysis, female and less obese participants showed higher HAV seropositive rates, whereas other characteristics such as age, alcohol use, smoking history, vaccine type, and follow-up duration were not associated with seropositivity (Table 1).

**Table 1:** Characteristics of study participants and related factors for seropositivity.

Characteristics	Distribution		Seropositivity	
	N	N/Total (%)	n	n/N (%)
Age at enrollment (years)			*p* = 0.797*	
17–19	303	68.1	231	76.2
20–22	135	30.3	102	75.6
23–28	7	1.6	5	71.4
Gender			*p*<0.001	
Male	295	66.3	204	69.2
Female	150	33.7	134	89.3
Obesity status			*p* = 0.052*	
Underweight	19	4.4	19	100.0
Normal	317	73.7	238	75.1
Overweight	61	14.2	45	73.8
Obese	33	7.7	22	66.7
Alcohol use			*p* = 0.192*	
None or low	63	14.4	45	71.4
Medium	36	8.2	34	94.4
High	106	24.2	87	82.1
Very high	233	53.2	165	70.8
Smoking history			*p* = 0.734	
Current or ex-smoker	26	5.9	19	73.1
Nonsmoker	417	94.1	317	76.0
Vaccination against hepatitis A			*p* = 0.598	
Havrix	222	49.9	171	77.0
Epaxal	223	50.1	167	74.9
Follow-up duration (months)			*p* = 0.131*	
20–21	160	36.0	114	71.3
22–23	78	17.5	62	79.5
24–25	207	46.5	162	78.3
Total	445	100.0	338	76.0

N, number of participants; n, number of participants who are seropositive.

*Calculated by linear by linear test.

In the multivariable analysis using a multiple logistic regression model, women and those categorized as medium alcohol use showed significantly higher seropositive rates compared with men and the groups with no or low alcohol use (Table 2).

**Table 2:** Related factors for seropositivity using a multiple logistic regression model*.

Characteristics	N	OR (95% CI)	*p* value
Age at enrollment (yrs)			
17–19	291	Reference	
20–22	130	1.07(0.65–1.77)	0.779
23–28	6	0.57(0.10–3.42)	0.539
Gender			
Male	289	Reference	
Female	138	3.57(1.90–6.72)	<0.001
Obesity status			
Underweight	19	Undefined†	0.998
Normal	311	Reference	
Overweight	61	1.26(0.66–2.40)	0.483
Obese	33	0.93(0.42–2.05)	0.849
Alcohol use			
None or low	60	Reference	
Medium	34	5.91(1.24–28.10)	0.026
High	105	1.85(0.85–4.01)	0.121
Very high	228	1.12(0.58–2.15)	0.732
Smoking			
Nonsmokers	402	Reference	
Smokers	25	1.34(0.52–3.45)	0.541
Vaccination against hepatitis A			
Havrix	212	Reference	
Epaxal	215	0.97(0.62–1.53)	0.896
Follow-up duration (months)			
20–21	155	Reference	
22–23	75	1.42(0.73–2.78)	0.306
24–25	197	1.43(0.87–2.35)	0.163

CI, confidence interval; N, number of participants: OR: odds ratio.

* Multiple logistic regression model included age (years), gender, BMI (kg/m^2^), AUDIT-K, smoking history, vaccine type, and follow-up duration (months) as covariates.

^†^ The OR is undefined because there was no seronegative participant in the relevant subgroup.

**Fig 1:**
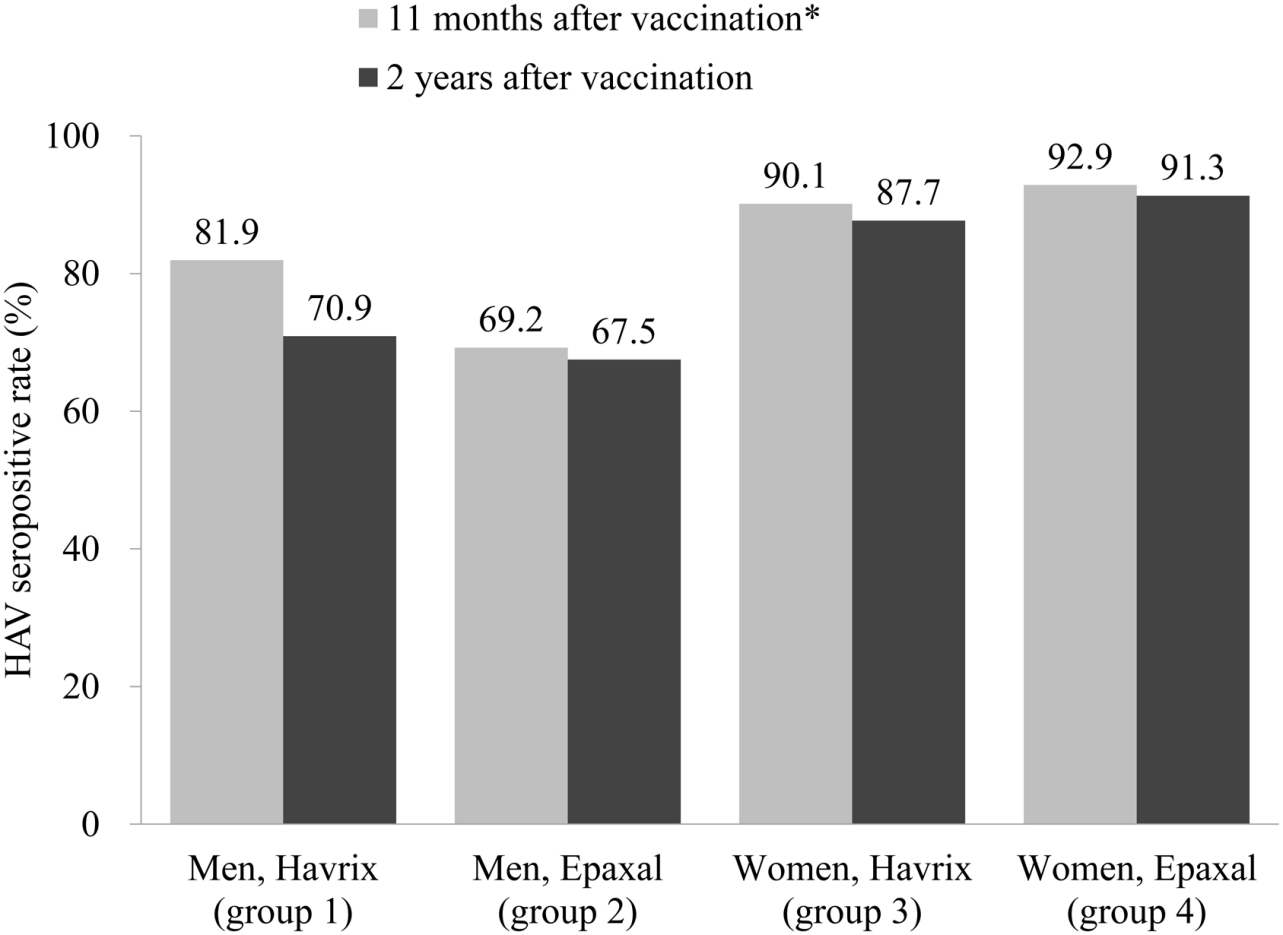
Change in the HAV seropositive rates from approximately 11 months to 2 years after vaccination. *Seropositive rates 11 months after a single-dose HAV vaccination were cited from our previous study [2].

We examined whether the effects of HAV vaccine type on seropositivity were different between men and women. The two vaccines yielded similar seropositive rates among male and female participants: 70.9% and 67.5% in men, and 87.7% and 91.3% in women, approximately 2 years after vaccination with Havrix and Epaxal, respectively (*p* for interaction = 0.304). In addition, the seropositive rate decreased substantially only in men vaccinated with Havrix (11.0% point) but decreased only <3% in men vaccinated with Epaxal and women vaccinated with Havrix or Epaxal from approximately 11 months to 2 years after vaccination (Fig 1).

## Discussion

Using a total serum anti-HAV antibody titer cut-off value of 20 IU/mL, the seropositive rate among young adults in this study was 77.0% and 74.9% approximately 2 years after a single-dose vaccination with Havrix and Epaxal, respectively.

Few studies have evaluated the seropositive rate approximately 2 years after a single-dose vaccination against HAV. In previous studies that included only adult participants and used 20 IU/mL as the cutoff value, the seropositive rates were 44.4%, 67.0%, and 97.8% at 9–29, 18–29, and 36 months after a single-dose vaccination against HAV, respectively [6–8]. These differences can be explained by the distinct gender ratios among the study participants. The female to male ratios were 1.41 and 1.17 in the studies that showed seropositivity rates of 97.8% and 67.0% 36 and 18–29 months after a single dose of HAV vaccination, respectively. From this perspective, our results (female to male ratio of 0.51 and seropositive rate of 76.0% 20–25 months after vaccination) were consistent with those of previous studies. If the gender ratio were 1.0, the seropositive rate would increase to 79.4%.

The comparison of the seropositivity approximately 2 years after a single-dose vaccination with Havrix and Epaxal indicates that the crude seropositive rates were 77.0% and 74.9%, respectively, whereas the gender-adjusted seropositive rates under the assumption of a gender ratio equivalent to 1.0 were 79.3% and 79.4% in the Havrix and Epaxal groups, respectively. Therefore, we can conclude that Havrix and Epaxal showed similar immunogenicity approximately 2 years after a single-dose vaccination.

We compared our current results with those obtained approximately 11 months after the vaccination, although the participants of the two studies were not identical. Among the 39 participants who dropped out from the study, 29 dropped out earlier than 11 months after the vaccination and nine dropped out later than 11 months after the vaccination. The female to male ratio was 5/24 (0.21) in the 29 early dropout participants and 2/7 (0.29) in the nine later dropout participants. The number of participants who dropped out after 11 months was relatively small, and the male to female ratios were similar in total participants evaluated at approximately 11 months and in those evaluated at 2 years after vaccination (0.50 and 0.51, respectively). Therefore, we think that the comparison of the results between the two time points is reasonable.

The interaction between gender and vaccine type previously observed approximately 11 months after vaccination was not observed approximately 2 years after a single-dose vaccination because the seropositive rate decreased substantially only in men vaccinated with Havrix, whereas the other 3 groups showed a minimal decrease in the seropositive rate. Therefore, even if higher immunogenicity with Havrix were consistently observed in men in the short term compared with Epaxal, vaccination of male adults with Havrix may have no advantage compared with Epaxal with respect to long-term immunogenicity.

We found that a high level of seropositivity was maintained up to approximately 2 years among women participants in this study. Further studies are needed to assess whether the seropositivity can be maintained beyond 2 years after single-dose HAV vaccination, because if so, a single-dose strategy may be more cost-effective, particularly in women.

In our study, only women and the medium alcohol use group showed significantly higher seropositive rates approximately 2 years after a single-dose vaccination in the multivariable analysis. Although low HAV immunogenicity in men has been consistently found in previous studies [6, 9–14], the dependence of HAV immunogenicity on demographic characteristics including alcohol use has not been studied. The seropositive rates in the groups with no or low alcohol use were not significantly different from those with high and very high alcohol use, and only the medium alcohol use group showed a significantly higher seropositive rate. However, further evaluation of the effect of alcohol use on HAV immunogenicity is needed because the participants had an extremely high level of alcohol use compared with Korean young adults and college students, and the sample size of this study was not large [15, 16].

The age distribution of the 38 participants who dropped out was similar to that of the final 445 participants, although their female to male ratio (0.14) was lower than that of the final participants (0.51). Because men showed lower seropositivity, the seropositive rate of all participants would have been lower than the result obtained if all of the participants, including the 38 who did not complete the study, were followed up. However, we think that the effect of those 38 dropouts on the total seropositivity may be minimal because their proportion was only 7.9%. More importantly, we think the dropouts would not decrease the internal validity of the association between seropositivity and vaccine type or demographic characteristics, which is the main result of this study.

In summary, the seropositive rate was 77.0% vs. 74.9% approximately 2 years after a single-dose vaccination with Havrix vs. Epaxal, respectively. Compared with the seropositive rate approximately 11 months after vaccination, only men vaccinated with Havrix showed a substantial decrease in the seropositive rate. Because the seropositive rate decreased by less than 3.0% 1 year after vaccination in all but the men vaccinated with Havrix, further studies are needed to assess whether these seropositive rates would be maintained in all groups more than 2 years after a single-dose HAV vaccination.

## Supporting Information

S1 Data Data including the measures of 445 participants underlying all the findings in Tables 1 and 2. (CSV)
